# Clinical Decision-Making Case: Non-Accidental Trauma

**DOI:** 10.21980/J8.53233

**Published:** 2025-12-31

**Authors:** H Michelle Greene, Anne P Runkle, Jennifer M Mitzman, Christopher E San Miguel, Krystin N Miller, Simiao Li-Sauerwine, Geremiha Emerson, Sorabh Khandelwal, Kelsey H Jordan, Jennifer Yee

**Affiliations:** * The Ohio State University College of Medicine, Department of Pediatrics, Columbus, Ohio; ^ The Ohio State University College of Medicine, Department of Emergency Medicine, Columbus, Ohio

## Abstract

**Audience:**

This clinical decision-making (CDM) case is intended for emergency medicine (EM) residents of all levels.

**Introduction:**

Non-accidental trauma (NAT) is a leading cause of morbidity and mortality in pediatrics. Every year in the United States, more than 656,000 children are found to be victims of NAT, causing over 1,800 deaths annually.^1^ Subtle abusive injuries are frequently missed in medical settings,^1^–^3^ and children may subsequently experience escalating or life-threatening abuse if interventions do not occur.^2^ Timely identification of abusive injuries in acute care settings is crucial to provide appropriate and potentially lifesaving care.

**Educational Objectives:**

By the end of this clinical decision-making case, learners will be able to: 1) demonstrate familiarity with the CDM case format and case play, 2) describe important historical information to obtain when suspecting non-accidental trauma, 3) recognize potential physical exam findings in non-accidental trauma, 4) justify appropriate diagnostic studies based on clinical findings and current evidence on occult injury in suspected pediatric abuse, and 5) propose an appropriate disposition plan for patients with non-accidental trauma.

**Educational Methods:**

This is a clinical decision-making boards case as outlined by the American Board of Emergency Medicine (ABEM). Each learner was paired with one instructor for the case, a scoring checklist by the instructor was used, and learners were given the opportunity to provide feedback after the case.

**Research Methods:**

Each CDM case session lasted approximately 20 minutes, with 15 minutes for the case and 5 minutes for debriefing and feedback. A 25-point critical action checklist was developed to evaluate each learner’s performance. Learners then provided verbal feedback on the cases to the examiners at the conclusion of their assessments.

**Results:**

Thirty-nine emergency medicine residents participated as learners for this clinical decision-making session, including 10 third-year residents, 12 second-year residents, and 17 first-year residents. Scoring checklists had a possible score of 25 points, with each point reflecting an equally weighted item. The average overall score was 16.85 of 25 possible points. Performance with respect to post-graduate year (PGY) is as follows: 18.0 for PGY-3s, 18.9 for PGY-2s, and 14.7 for PGY-1s. One resident had a perfect score of 25/25. There was no threshold passing score; therefore, no one resident “failed” this mock structured interview.

**Discussion:**

Performance of our learners varied and unexpectedly, our second-year residents outperformed our third-year residents. We believe this is due to our PGY-2 learners being responsible for the primary care of stroke patients in our department, which makes their identification of head bleed likely more recently retrievable. We reviewed outlier items (where residents all scored very high or if the score was lower compared to other items) to determine if this is an appropriately written item, and if so, we will use lower-scoring items as learning opportunities to emphasize within future didactics sessions. One of these items involved asking who the patients’ caregivers were, which may be attributed to being unaware of the relevance of this question to NAT, or, more likely, that NAT was not a top differential diagnosis in the early aspects of the case.

Verbal feedback from our learners primarily focused on widening our accepted differential diagnoses for future iterations. We believe that this case is appropriate for all levels of learning, particularly when a formative approach (assessment for learning) is used. Given the feedback learners and instructors provided, we believe this case has high value impact in reviewing high-risk or high-acuity pediatric pathology.

**Topics:**

Emergency medicine, pediatrics, non-accidental trauma, pediatric head trauma, head bleed.

## USER GUIDE

**Table:** 

List of Resources:	
Abstract	292
User Guide	294
For Examiner Only	296
Certifying Exam Assessment	302
Stimulus	304
Debriefing and Evaluation Pearls	310

**Learner Audience:**

Interns, Junior Residents, Senior Residents

**Time Required for Implementation:**

Case: Clinical Decision-Making cases are 15 minutes as directed by American Board of Emergency Medicine (ABEM).

Debriefing: 5–10 minutes per case.

**Recommended number of learners per instructor:**

We recommend that the instructors facilitate the case one-on-one with learners. We had a mock exam day where multiple facilitators ran the same case repeatedly with different residents.

**Topics:**

Emergency medicine, pediatrics, non-accidental trauma, pediatric head trauma, head bleed.

**Objectives:**

By the end of this CDM case, learners will be able to:

1. Demonstrate familiarity with the CDM case format and case play
2. Describe important historical information to obtain when suspecting non-accidental trauma
3. Recognize potential physical exam findings in non-accidental trauma
4. Justify appropriate diagnostic studies based on clinical findings and current evidence on occult injury in suspected pediatric abuse
5. Propose an appropriate disposition plan for patients with non-accidental trauma

### Linked objectives, methods and results

By participating in this clinical decision-making case, residents have the opportunity to practice assessing a non-accidental trauma patient with critical physical exam findings (objectives 1–3). Residents will be evaluated on their appropriate management and disposition (objectives 4–5) of their patient. Additionally, a built-in debriefing session at the end of the case provided real-time feedback to learners to ensure an “assessment for learning” component to the exercise.

### Recommended pre-reading for instructor

- American Board of Emergency Medicine. Sample Case: Clinical Decision Making. YouTube. Published November 27, 2024. Accessed November 23, 2025. https://www.youtube.com/watch?v=Dv_ga0Ei7oY
- American Board of Emergency Medicine Certifying Exam Case Materials: Clinical Decision Making. Published November 2024. Accessed November 23, 2025. Case-Materials_Clinical-Decision-Making.pdf
- Christian CW. The evaluation of suspected child physical abuse. *Pediatrics.* 2015 May;135(5):e1337–54. doi:10.1542/peds.2015-0356. Erratum in: Pediatrics. 2015 Sep;136(3):583. doi:10.1542/peds.2015-2010
- Letson MM, Cooper JN, Deans KJ, et al. Prior opportunities to identify abuse in children with abusive head trauma. *Child Abuse Neg*l. 2016 Oct;60:36–45. Epub 2016 Sep 25. doi:10.1016/j.chiabu.2016.09.001
- Harper NS, Feldman KW, Sugar NF, Anderst JD, Lindberg DM. Examining siblings to recognize abuse investigators. Additional injuries in young infants with concern for abuse and apparently isolated bruises. *J Pediatr*. 2014 Aug;165(2):383–388.e1. https://doi.org/10.1016/j.jpeds.2014.04.004
- Haney S, Scherl S, DiMeglio L, Perez-Rossello J, Servaes S, Merchant N. Evaluating young children with fractures for child abuse: Clinical report. *Pediatrics*. 2025 Feb. 1;155(2):e2024070074. https://doi.org/10.1542/peds.2024-070074
- Lindberg D, Makoroff K, Harper N, et al. Utility of hepatic transaminases to recognize abuse in children. *Pediatrics*. 2009;124(2):509–516. doi:10.1542/peds.2008-2348

### Results and tips for successful implementation

This clinical decision-making case was created as a collaboration between Emergency Medicine (EM) faculty and Pediatric Emergency Medicine (PEM) faculty including one member who completed a Child Abuse Pediatrics Fellowship. Based on feedback provided by our learners and instructors, this case was best implemented as a CDM case (or what was formerly known as a structured interview). All 39 learners were EM residents (10 third-year residents, 12 second-year residents, and 17 first-year residents). Each learner was allotted 15 minutes for the case and 5 minutes for a debriefing afterwards. Instructors graded each learner on a 25-point grading sheet. Learners provided verbal feedback to their case facilitators during the debriefing period. Since original pilot data, the questions listed here were made more specific and updated to better reflect the new Certifying Exam based on evolving ABEM information. The average score for all learners was 16.85 out of 25 total items. The averages for each post graduate year (PGY) class were as follows: 18.0 for PGY-3s, 18.9 for PGY-2s, and 14.7 for PGY-1s. This numerical data represents our institutional iteration of our initial understanding of the Clinical Decision-making Cases; therefore, we only included one rationale prompt in the History section, and our Transition of Care section did not include “Provides sign-out.”

We updated our initial 25-point grading sheet from our original assessment to include an additional rationale prompt for the History section and “sign out” action for Transition of Care section in this manuscript. Other changes made to this case for subsequent use included: expanded differential diagnoses, clarification edits to one historical question, and we added a social work consult as a potential mandated reporter under “treatment and other actions.”

Feedback from our faculty prompted us to include an abdominal exam, pulse palpation and capillary refill to the total physical exam. We also found that resident differential diagnoses at times included both “non-accidental trauma” and “accidental trauma,” so if the resident stated “accidental trauma,” faculty were then prompted to ask the resident to be more specific.

With respect to the computed tomography (CT) image linked to this case, a reading of subdural hematoma and/or subarachnoid hemorrhage was not provided, and instead residents were expected to interpret the image themselves. We noticed multiple residents struggled to identify these critical findings, many of which were first-year level of training. When incorporating this case at your institution, instructors may want to consider adding a radiology read for first year resident learners or finding an image that is less subtle.

We included the liver function tests (LFTs) as an item on the grading sheet in order to highlight that this is a decision point to determine whether or not to obtain a CT of the abdomen/pelvis in pediatric non-accidental trauma. With elevated liver function tests or abdominal bruising, distention, or tenderness, a CT of the abdomen/pelvis should be considered.^5^

## Figures and Tables

**Table:** 

**I. History**		Yes	No
**1a**	Ask who the patient’s caregivers are		
**1b**	Ask if the patient’s vomiting was bilious		
**1c**	Ask if the patient received vitamin K		
**2**	*Vitamin K administration:* to assess bleeding risk		
**II. Physical Examination**			
**3a**	Fully undressed skin exam		
**3b**	Fontanelle exam		
**3c**	Intra-oral exam		
**III. Broad Concepts: Intra-oral exam**			
**4**	To evaluate for frenulum injuries		
**IV. Differential Diagnosis**			
**5a**	Non-accidental trauma		
**5b**	Intracranial hemorrhage		
**5c**	Others: intussusception, bleeding diatheses/disorder, sepsis, or pathologies related to intractable crying		
**V. Diagnostic Studies**			
**6a**	Skeletal survey		
**6b**	LFTs		
**6c**	CT head		
**7**	LFTs can help determine need for CT abdomen/pelvis in pediatric non-accidental trauma		
**8**	CT head to evaluate for skull fracture or ICH		
**VI. Treatment and Other Actions**			
**9a**	Child protective services/social work		
**9b**	Consult neurosurgery		
**10**	Mandated reporting for concern for NAT		
**11**	Traumatic ICH to evaluate need for surgical intervention		
**VII. Final Diagnosis**			
**12**	Non-accidental trauma with ICH (must include both components)		
**VIII. Disposition**			
**13**	Admit to the PICU		
**14**	For frequent neurologic examinations		
**IX. Transitions of Care**			
**15a**	Explain diagnosis to the patient’s father		
**15b**	Describe neurologic exam findings during handoff		

**Table:** 

**Candidate Task Sheet**	
**#1**	**Emergency Department Admitting Form**
**#2**	**Additional Historical Information**
**#3**	**Physical Exam Findings**
**#4**	**Diagnostic Studies**

**Table:** Clinical Decision-Making Task Sheet

**CASE PARAMETERS**
This is a 15-minute case You will interact with two examiners. This is an interview style without role playing; you should simply reply to the questions asked. You may be interrupted to move you through the case; this is not a reflection of your performance.
**PATIENT INFORMATION**
Patient presents for excessive crying for the past few hours.
**VITAL SIGNS**
BP: 90/58 P: 112 beats per minute R: 32 respirations per minute T: 37° Celsius 02Sat: 100% room air
**TASK STATEMENT**
Your tasks are as follows: List pertinent elements of a focused history and physical exam Develop an appropriate differential and/or provisional diagnosis Select and interpret appropriate studies Articulate appropriate patient management including discharge instructions

**STIMULUS 1:** Emergency Department Admitting Form

**Patient Information**	
**Patient Name**	Sara Smith
**Age**	12-weeks-old
**Gender**	female
**Method of Arrival**	Private vehicle
**General Appearance/History of Present Illness**	Patient presents for excessive crying for the past few hours. She is crying in father’s arms, inconsolable.
**Vital Signs on Initial Presentation**	BP: 90/58 P: 112 beats per minute R: 32 respirations per minute T: 37° Celsius O2 sat: 100% on room air

**STIMULUS 2:** Historical Information

**History of Present Illness/Description of Event**	
Patient’s father has brought her into the emergency department for increased fussiness for the past week. She was born via a spontaneous vaginal delivery at 39 weeks without pregnancy or birth complications. She has had two episodes of non-bloody, non-bilious emesis over the past 24 hours, one associated with feeds and one without. Her dad reports that she has been taking in one less bottle of formula per day but otherwise has had a normal number of wet diapers and the normal amount of stools. Patient’s father denies measured fever, cough, congestion, diarrhea, or blood in the stool.	
**History**	
**Past Medical History**	Healthy. She was born via a spontaneous vaginal delivery at 39 weeks without pregnancy or birth complications. She has received all of her vaccinations from birth until now, including vitamin K vaccine and hepatitis B vaccine at birth.
**Past Surgical History**	none
**Medications**	none
**Allergies**	none
**Social History**	She is an only child who lives at home with her parents and attends an in-home daycare

**STIMULUS 3:** Physical Exam Findings

**Physical Examination**	
**General**	Patient is inconsolable, held by father.
**Dermatologic**	No bruises or hematomas.
**HEENT**	Generalized swelling to the right side of her temporoparietal scalp. No palpable skull defects or mastoid bruising. Her fontanelle is flat without subconjunctival hemorrhages or periorbital ecchymosis. Small nonbleeding inferior labral frenulum hematoma is present.
**Neck**	normal
**Respiratory**	normal
**Cardiac**	normal
**Abdominal**	normal
**Extremities**	There are no hair tourniquets, bony deformities, or tenderness to palpation.
**Neurologic**	Glascow Coma Scale is 13 (E43M6). She is moving all four extremities without difficulty.

**STIMULUS 4:** Diagnostic Studies

**CBC**	normal
**CMP**	normal
**Glucose**	normal
**Lipase**	normal
**CT head** 9	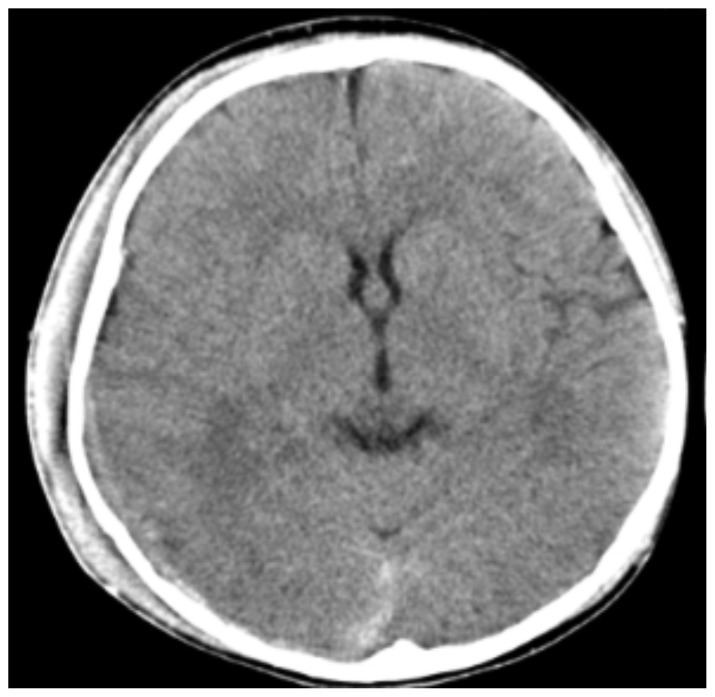

## FOR EXAMINER ONLY

### Clinical Decision-Making Case: Non-Accidental Trauma Summary

**Diagnosis:** Non-accidental trauma

**Case Summary:** The patient is a 12-week-old female who presents with excessive crying for several hours.

Patient’s father has brought her into the emergency department for increased fussiness to the past week. She was born via a spontaneous vaginal delivery at 39 weeks without pregnancy or birth complications. She is an only child who lives at home with her parents and attends an in-home daycare. She has had two episodes of non-bloody, non-bilious emesis over the past 24 hours, one associated with feeds and one without. Her dad reports that she has been taking in one less bottle of formula per day but otherwise has had a normal number of wet diapers and the normal amount of stools. Patient’s father denies measured fever, cough, congestion,, diarrhea, or blood in the stool. She has received all of her vaccinations from birth until now, including vitamin K vaccine and hepatitis B vaccine at birth.

### Clinical Decision-Making Case: Non-Accidental Trauma Examiner Script

**Case Introduction:**

“Hello Doctor, this is a clinical decision-making case. There is no role playing. In response to the questions I will ask, please give me a LIST of information you would gather to come to a final diagnosis. At times, I may interrupt you to move you through the case; this is not a reflection of your performance. You will have 15 minutes to complete the case. Before we begin, do you have any questions?”

“Your patient is a 12-week-old female who presents with excessive crying for several hours.”

Provide Learner Stimulus #1

#### HISTORY

Prompt 1:

“Here is the initial information regarding this patient. After you have read it, please give me a list of the additional historical information you would obtain.”

Scoring Guidelines:

Rationale: Learners should ask who the caregivers are, if the vomiting is bilious, and whether the patient received vaccines (vitamin K) at birth.

Prompt 2a:

“Why might it be important to ask about a pediatric patient’s vaccination history?”

Scoring Guidelines:

Rationale: To assess spontaneous bleeding risk for those who did not receive vitamin K administration at birth. Late vitamin K-deficient bleeding can occur one week to six months after birth, particularly in exclusively breastfed infants who did not receive vitamin K prophylaxis. The highest risk peaks between two and eight weeks of life.^8^

Prompt 2b:

“How could asking about a pediatric patient’s caregivers be helpful to you?”

Scoring Guidelines:

Rationale: Learners should express the importance of obtaining a full social history, to assess potential for non-accidental trauma, and to potentially obtain further collateral history.

#### PHYSICAL EXAMINATION

“Thank you, doctor, here is the additional historical information.”

Provide Learner Stimulus #2 and read pertinent information.

Prompt 3:

“Now would you please discuss what you will be assessing on the physical exam of this patient.”

Note for examiner: if any exam stated is not specific enough you can ask the learner to clarify their exam.

Scoring Guidelines:

Rationale: Learners should list at minimum undressing the patient for a full skin exam, fontanelle exam, and intra-oral exam.

Prompt 4:

“Doctor, why would an intra-oral exam be important in this case?”

Scoring Guidelines:

Rationale: Intra-oral exam allows for evaluation of frenulum injuries.

“Thank you, doctor, here are the physical exam findings.”

Provide Learner Stimulus #3 and read pertinent information.

#### DIFFERENTIAL DIAGNOSIS

Prompt 5:

“Based on what you now know, what are the top three items on your differential diagnosis based on the most likely conditions?” (If more than three conditions are mentioned, say, “OK, thank you. Please give me your three, and only three, most likely diagnoses.”)

Scoring Guidelines:

Rationale: Non-accidental trauma and intracranial hemorrhage MUST be mentioned, additional differentials can include intussusception, bleeding diatheses/disorder, sepsis, or other pathologies related to intractable crying.

#### DIAGNOSTIC STUDIES

Prompt 6:

“Based on what you know and your working differential diagnosis, what, if any, diagnostic studies would you order?”

Scoring Guidelines: Learners should at minimum express desire to order a skeletal survey, liver function tests (LFTs), and a CT head.

Prompt 7:

“Doctor, not necessarily specific to this patient, but in any infant presenting with possible non-accidental trauma, why would LFTs be important?

Scoring Guidelines:

Rationale: To assess potential need for a CT abdomen to evaluate for traumatic intra-abdominal injuries. Abdominal injury has been identified in 3.2% of potentially abused children; it is not so common to recommend universal imaging, but it still may be associated with significant morbidity and mortality. Pediatric abdominal injuries may also be difficult to recognize clinically. Using a laboratory screening threshold of 80 IU for transaminase levels may allow clinicians to detect occult abdominal injury with a sensitivity/specificity of 77% and 82%, respectively.^7^

Prompt 8:

“Doctor, in a patient with concern for non-accidental trauma, what specifically would you be looking for on a head CT (computed tomography)?

Scoring Guidelines:

Rationale: CT head would allow clinicians to evaluate for skull fractures or intracranial hemorrhage (ICH).

Provide Learner Stimulus #4: Complete Blood Count (CBC), Basic Metabolic Panel (BMP), LFTs, lipase, CT head.

#### TREATMENT AND OTHER ACTIONS

Prompt 9:

“Doctor, could you please interpret this CT head image for me?”

Prompt 10:

“Based on what you now know, what treatments, if any, would you order and/or what actions, if any, would you perform?”

Scoring Guidelines:

Rationale: Learner at minimum should express desire to consult child protective services/social work and neurosurgery. Neurosurgery should be consulted to evaluate for need for procedural or operative intervention, to recommend frequency of neurologic assessments, and to assess the need for repeat head imaging.

Prompt 11:

“Doctor, why would it be important to consult child protective services/social work in this case?”

Scoring Guidelines:

Rationale:

Social work and/or child protective services should be contacted for mandated reporting of concern for non-accidental trauma.

#### FINAL DIAGNOSIS

Prompt 12:

“Based on everything you know about this case, what is your final diagnosis?”

Scoring Guidelines:

Rationale: Non-accidental trauma with ICH (needs to include both components). Verbalizing “subdural hematoma” and/or “subarachnoid hemorrhage” meets the critical action. If the candidate mentions something vague such as “trauma” or “NAT” (usually a physical exam finding or imaging finding instead of diagnosis), examiner asks, “can you be more specific about the diagnosis?”

#### DISPOSITION

Prompt 13:

“Based on what you know, what should be the disposition of this patient?”

Prompt 14:

“Why would you [admit/discharge] this patient?”

Scoring Guidelines:

Rationale: Learner should express that the patient requires admission to the Pediatric Intensive Care Unit (PICU) for frequent neurologic checks and for further Child Protective Services (CPS) investigation.

Scoring Guidelines:

Verbalizing that the patient should be admitted to PICU for serial neurologic examinations and further CPS investigation.

#### TRANSITION OF CARE

Prompt 15:

“What physical exam findings would it be important to communicate to the team taking over the case?”

Scoring Guidelines:

Rationale: Describing the patient’s neurologic exam findings during handoff allows for the team to assess for any changes on subsequent neurologic checks.

*Thank you, Doctor. That concludes this case.*

*Please tear up your notes.*

## CERTIFYING EXAM ASSESSMENT

*Clinical Decision-Making Case: Non-Accidental Trauma*

Learner: _________________________________________

**Summative and formative comments:**

## DEBRIEFING AND EVALUATION PEARLS

### Clinical Decision-Making Case: Non-Accidental Trauma

#### History

- Child physical abuse can be subtle, especially if there is not a history of injury given to the medical provider.
- Fussiness, vomiting, and altered mental status can signal a possible intracranial injury but can be easy to dismiss in an infant or nonverbal patient.
- Examinations for infants, particularly nonmobile infants, should include a full skin exam and frenulum exam, regardless of chief complaint.
- If there are injuries on examination, ask the family open-ended questions such as, “tell me about this.” or “what happened here?”
- Ascertaining who was present during an injury, when the injury occurred, and when the child was last in their normal state of health are all important.
- If there was a delay in seeking care, ask about the reasoning for the delay.

#### Exam

- Young infants who cannot crawl or walk should not be able to self-inflict injuries such as bruises, frenulum tears, or subconjunctival hemorrhages.
- TEN4-FACESp is a useful acronym to remember abnormal bruising locations in children four years and under.^4^
  - Bruising to **T**orso, **E**ars, **N**eck, **F**renulum, **A**ngle of the jaw, **C**heeks, **E**yelids or **S**ubconjunctivae
  - Infants **4.99** months and younger with any bruise, anywhere
  - **P**atterned bruising

#### Assessment and Plan

- Do not assume the caregiver accompanying a child is the perpetrator. You are not accusing anyone of abuse or performing a criminal investigation. Your job is to determine medically if the history makes sense for the injuries, if there are additional occult injuries, and to look for other possible medical causes of the patient’s findings.
- The standard workup for children with suspected physical abuse includes a skeletal survey (under 2–3 years of age),^5^ head CT (under 6–12 months or if signs of intracranial injury),^6^ abdominal labs (AST, ALT, lipase under age 5 years).^7^
- In suspected child abuse, AST or ALT >80 units/liter or an elevated lipase indicate a CT of the abdomen and pelvis with contrast. If a child has abdominal bruising, they should have a CT of the abdomen and pelvis with contrast, regardless of lab values.^7^
- Suspected child abuse should be reported to Child Protective Services and possibly Law Enforcement—you do not need to prove abuse happened.
- If you do not have social work at your institution, you may have to be the one calling Child Protective Services (CPS) yourself. You do not have to know the answer to every question they ask.
- If your institute does not have capabilities for skeletal survey, inpatient pediatric services, or CPS is not available to plan a safe discharge, you may need to transfer a patient to another facility.

## References

[1] Christian CW (2015). The evaluation of suspected child physical abuse. *Pediatrics*.

[2] Letson MM, Cooper JN, Deans KJ (2016). Prior opportunities to identify abuse in children with abusive head trauma. *Child Abuse Negl*.

[3] Harper NS, Feldman KW, Sugar NF, Anderst JD, Lindberg DM (2014). Examining siblings to recognize abuse investigators. Additional injuries in young infants with concern for abuse and apparently isolated bruises. *J Pediatr*.

[4] Pierce MC, Kaczor K, Lorenz DJ (2021). Validation of a clinical decision rule to predict abuse in young children based on bruising characteristics. JAMA Netw Open.

[5] Lindberg DM, Berger RP, Reynolds MS, Alwan RM, Harper NS (2014). Examining siblings to recognize abuse investigators. Yield of skeletal survey by age in children referred to abuse specialists. *J Pediatr*.

[7] Wootton-Gorges SL, Soares BP, Alazraki AL (2017). ACR Appropriateness Criteria^®^ Suspected Physical Abuse-Child. *J Am Coll Radiol*.

[8] Lindberg D, Makoroff K, Harper N (2009). Utility of hepatic transaminases to recognize abuse in children. *Pediatrics*.

[9] Perrone S, Beretta V, Petrolini C, Scarpa E, De Bernardo G, Buonocore G Late vitamin k deficiency bleeding in infancy: The time to ensure effective prevention. Nutr Rev.

[10] Radiology Cases of Subdural Hematoma Basal cell carcinoma on the forehead [clinical image]. Pediatric Imaging.

